# Seroprevalence of *Ehrlichia canis* in Clinically Suspect Dogs and Its Association with Clinical and Social Factors in Urban and Rural Areas of North-Central Mexico

**DOI:** 10.3390/vetsci12080771

**Published:** 2025-08-18

**Authors:** Mario Cuauhtémoc Cárdenas-Arias, Rafael Rodríguez-Venegas, Pedro Antonio Robles-Trillo, Francisco Gerardo Véliz-Deras, Alan Sebastián Alvarado-Espino, Vicente Homero González-Álvarez, Martín Alfredo Legarreta-González, Rafael Rodríguez-Martínez

**Affiliations:** 1 Programa de Maestría en Ciencias en Producción Agropecuaria, Universidad Autónoma Agraria Antonio Narro, Unidad Laguna, Torreón 27054, Mexico; cuatecardenas_2211@hotmail.com; 2 Departamento de Ciencias Médico Veterinarias, Universidad Autónoma Agraria Antonio Narro, Unidad Laguna, Torreón 27054, Mexico; rafar.v.v@gmail.com (R.R.-V.); velizderas@gmail.com (F.G.V.-D.); 3 Departamento de Producción Animal, Universidad Autónoma Agraria Antonio Narro, Unidad Laguna, Torreón 27054, Mexico; parobles58@gmail.com (P.A.R.-T.); alanalvaes@gmail.com (A.S.A.-E.); 4 Facultad de Medicina Veterinaria y Zootecnia No. 2, Universidad Autónoma de Guerrero, Carretera Federal Acapulco–Pinotepa Nacional s/n, Cuajinicuilapa 41940, Mexico; homero.uagro@gmail.com; 5 Universidad Tecnológica de la Tarahumara, Carretera Guachochi-Yoquivo, Guachochi 33187, Mexico; 6 Posgraduate Department, Fatima Campus, University of Makeni (UniMak), Makeni City 00232, Sierra Leone

**Keywords:** Rickettsia, immunochromatography, epidemiology, haemoparasite, zoonosis, multiple correspondence analysis, logistic regression

## Abstract

The objective of this study was to estimate the seroprevalence of *Ehrlichia canis* in clinically suspect dogs in the Laguna Region of Coahuila, Mexico, and to analyse its association with several clinical, haematological, and social variables. The present study focuses on the veterinary care provided to a total of 249 dogs during the period from August 2023 to November 2024. The implementation of an immunochromatographic test was undertaken for the purpose of antibody detection. The observed seroprevalence was 63.9% (*n* = 156). Firstly, statistically significant associations were identified with clinical haemorrhagic signs (77.33%; OR = 5.03; *p* < 0.001), anaemia (81.25%; OR = 4.17; *p* < 0.001), and thrombocytopenia (96.26%; OR = 43.24; *p* < 0.001). Secondly, rural dogs exhibited a higher prevalence (70.54%) compared to urban dogs (54.17%), which was found to be statistically significant (*p* < 0.01). Multivariate analysis and logistic regression identified thrombocytopenia as the most robust predictor. The findings emphasise the substantial circulation of *Ehrlichia canis* in the region, thereby reinforcing the clinical significance of haemorrhagic manifestations and haematological parameters as diagnostic tools in low-resource settings. The implementation of epidemiological surveillance, vector control, and community education programmes is recommended in order to reduce the impact of this disease on animal and public health.

## Simple Summary

Canine Ehrlichiosis, an illness caused by the bacterium *Ehrlichia canis*, can present with symptoms including fever, haemorrhages, anaemia, and a reduction in the animal’s immune system. The present study analysed the presence of the aforementioned disease in 249 clinically suspect dogs in the Laguna Region of Coahuila, Mexico, between 2023 and 2024. The most prevalent signs were bleeding, anaemia, and thrombocytopenia, the latter being the strongest indicator. Furthermore, it was determined that canines residing in rural areas are more susceptible to infection compared to their urban counterparts.

## 1. Introduction

The increase in global temperature experienced in recent decades has led to abrupt variations in temperature, precipitation and flooding patterns [1,2,3,4] as well as created environments conducive to the proliferation of disease vectors, particularly ticks [5,6,7] and mosquitoes [8]. This phenomenon has been linked to an increase in tick-borne diseases in specific regions worldwide [9,10,11], including developed countries [12,13,14,15]. The destruction of the vectors’ ecological niches due to environmental change has resulted in their migration and expansion [2,13,16,17].

The emergence and re-emergence of zoonotic diseases poses a significant threat to global public health [3,10,18,19,20,21,22]. Presently, there is a paucity of information regarding zoonotic diseases spreading in the Americas, which is a significant challenge, given that these pathologies represent a serious threat to the region [23,24,25,26]. Among the threats identified are rickettsioses, caused by several species of Rickettsia, and zoonotic diseases transmitted by bites of arthropods such as ticks, lice, mites, and fleas [17,27,28,29,30,31]. Its epidemiology has historically been linked to a cycle composed of interaction between the reservoir, the arthropod, and the vector, as well as humans [1,30,32,33]. The brown dog tick (*Rhipicephalus sanguineus*) is a biological vector of ehrlichiosis, with canids and felids being its main host [4,27,28,31,32,34,35,36,37,38,39]. In addition, it has been demonstrated to facilitate the transmission of babesiosis, hepatozoonosis, and rickettsiosis [29,33].

In Mexico, research on zoonotic diseases, particularly ehrlichiosis, is extremely limited and underreported. The extant literature on this subject is predominantly confined to a few urban centres [3,19,40,41]. Furthermore, there is a paucity of analysis regarding the association between the prevalence of the disease and specific characteristics or variables of the population (both owners and pets) that could be determining elements in order to ascertain its distribution, establish possible risk groups [17,40], and serve to establish specific prevention and control measures in the areas identified for disease management [2,9,10,14,19,28,34,42], considering that domestic dogs are the main transmitters of the disease [43] and that their proximity to humans makes them an essential factor in potential transmission [18,40,44]. In view of the findings outlined above, a seroprevalence study was conducted in the Laguna Region of the State of Coahuila, Mexico, with the objective of ascertaining the prevalence of *Ehrlichia canis*. The present study also sought to elucidate its association with various social, public health, and clinical haematological risk factors that are of veterinary medical significance.

## 2. Materials and Methods

The Laguna de Coahuila Region, located in north-central Mexico (25°32′40′ N, 103°26′31′ W), is distinguished by its arid climate, characterised by limited water resources and high temperatures during summer months (reaching 45.3 °C) and low temperatures during winter (ranging from 0 °C to 8 °C). The region experiences an average annual rainfall of 324 mm, with a maximum altitude of 3000 m above sea level. The region in question comprises five municipalities: Torreón, Francisco I. Madero, Viesca, San Pedro de las Colonias, and Matamoros.

For the study, a blood analysis was carried out on 249 dogs that were clinically treated by jugular or cephalic venipuncture, and whose specimens were collected between August 2023 and November 2024, using vacutainer tubes with anticoagulant (EDTA). Immediately following collection, the blood samples were analysed by a lateral flow immunochromatographic assay (Uranotest snapquattro, Uranovet, Barcelona, Spain; sensitivity 95%; specificity 94.6%) and subsequently tested for red and white series (BC-30 Vet, Mindray, Seoul, Republic of Korea).

Variables associated with the owner were analysed, including the following: geographical location (rural = 1; urban = 0); highest level of schooling (no schooling, elementary, middle, high school, or higher); and municipality of owner’s residence (Francisco I. Madero, San Pedro, Matamoros or Viesca). Variables associated with the dog were included, as follows: age (puppy <1 year = 1; adult ≥1 year = 0); ticks (presence = 1, absence = 0); haemorrhage (-any type of- presence- = 1, absence = 0); anaemia (-HCT <32.5%- presence = 1, absence = 0); thrombocytopenia (-PLT < 117 × 10^9^/L- presence = 1, absence = 0); leukopenia (-WBC < 6 × 10^9^/L- presence = 1, absence = 0); and lymphopenia (-LYM < 0.8 × 10^9^/L- presence = 1, absence 0).

Prior to commencing the sampling process, a comprehensive calculation of the sample size was conducted, along with an estimation of the true prevalence of the disease. These calculations were performed utilising the epidemiological software WinEpi2.0. In order to assess the association between the prevalence of canine ehrlichiosis and the variables analysed, a logistic regression model was implemented. The calculation of probabilities of succession of events was performed using the odds ratio (OR) and contingency tables to obtain the chi-squared (χ^2^) distribution. To this end, a multiple correspondence analysis (MCA) was implemented to identify variables that contribute significantly to the variability observed in the survey instrument [45]. Subsequently, a selection of the variables that exhibited the most significant explanatory value in the model, such as thrombocytopenia and diagnosis, was carried out, and an ordinal logistic regression incorporating another variable, platelet count, was performed. The statistical analysis was conducted utilising R software, version 4.4.1 [46], and a statistical significance level of *p* < 0.05 was considered.

## 3. Results

Of the 249 samples that were analysed, 156 were found to be positive for *Ehrlichia canis*, which corresponds to an actual prevalence of 63.9%, when the sensitivity and specificity of the test used are taken into account. Conversely, the apparent prevalence, that is to say, the presence of the disease in the region irrespective of the tests utilised, was 62.65%.

In the descriptive statistical analysis of the non-dichotomous variables that constitute the social risk factors and public health of the study subjects (see Table 1), it is evident that only the variable ‘origin of the sample’ exhibits statistical significance (*p* < 0.05). The highest number of samples was obtained in the cities of Matamoros and San Pedro (93 each), and the highest number of positive patients was identified in Matamoros (58). However, the highest prevalence was recorded in the city of Francisco I. Madero with 83.02%. The analysis of the data revealed that there were no significant differences in the educational levels of the owners of the sampled dogs (*p* > 0.05).

The results of the dichotomous variables (Table 2) demonstrate a statistical difference (*p* < 0.01) in three of the clinical variables analysed with regard to the prevalence of *Ehrlichia canis*. The following indicators were considered in the present study: the presence or absence of bleeding, anaemia, and thrombocytopenia. With regard to the presence or absence of haemorrhages, of the 249 dogs sampled, 150 showed signs of haemorrhages, and of these, 116 were positive for *Ehrlichia canis*, while, of the 99 that did not show signs of haemorrhages, 40 were positive for *Ehrlichia canis*. This finding demonstrates a prevalence of 77.33% in dogs affected by haemorrhages, in contrast to the 40.4% observed in those that did not show haemorrhages. In relation to the canines affected by anaemia, 96 of the 249 dogs tested exhibited signs of anaemia, of which, 78 (81.25%) were positive for *Ehrlichia canis*. Conversely, within the cohort of 153 animals that did not exhibit signs of anaemia, 78 (50.98%), with an an odds ratio (OR) of 4.16, were found to be positive for the same organism. Furthermore, 107 of the 249 canine specimens examined demonstrated thrombocytopenia, of which, 103 were positive for *Ehrlichia canis* (96.26%), while of the 142 specimens not showing thrombocytopenia, 53 were positive for *Ehrlichia canis* (37.32%), OR de 43.24. Secondly, statistically significant differences (*p* < 0.01) were observed for the variable ‘owner’s residence’, with a higher prevalence of *Ehrlichia canis* in dogs of owners residing in rural areas compared to those residing in urban areas (70.54% vs. 54.17%, respectively) with an OR for the relationship between seroprevalence and the rural residence variable of 2.02.

**Figure 1:**
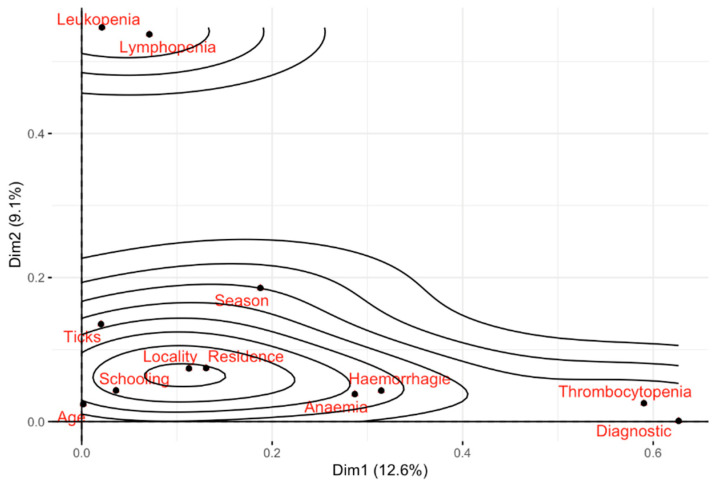
Discrimination and association measures from multiple correspondence analysis.

The multiple correlation analysis demonstrates that dimension 1 (Dim1) accounts for 12.6% of the variance of the model, while dimension 2 (Dim2) explains 9.1%, thus indicating that both dimensions collectively contribute to an explanation of 21.73%. The graphical representation of this analysis is presented in Figure 1, which illustrates that the model exhibits the strongest explanatory association with the variables thrombocytopenia and diagnosis.

**Figure 2:**
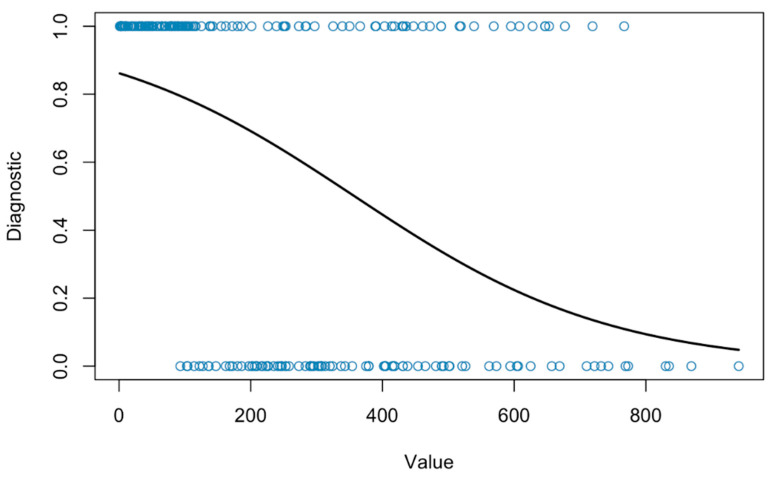
Logistic regression between the variables ‘Diagnostic’ and platelet value (10^9^/L)(Value).

Subsequent to the establishment of an association between the most significant dichotomous variables (thrombocytopenia and diagnosis), a logistic regression analysis was conducted, on this occasion between these two variables and the platelet value identified in the study (Value). As demonstrated in Figure 2, a negative association is evident between platelet count and the probability of a positive diagnosis for *Ehrlichia canis*.

## 4. Discussion

### 4.1. Ehrlichia canis Seroprevalence

The objective of this study was to ascertain the seroprevalence of *Ehrlichia canis* in the Laguna Region of the State of Coahuila, Mexico, and to identify its association with various social, public health, and clinical haematological risk factors of veterinary-medical interest. With regard to seroprevalence, the results obtained indicate an active and sustained circulation of *Ehrlichia canis* in the canine population of the region, with the presence of associated clinical signs. This value is considerably high (63.9% prevalence) compared to other regions of the country, such as the southeast (44%), the northwest (21–49%) [25], and the city of Mérida, Yucatán (44%), but lower than that obtained in the states of Guerrero (77.1%) and Morelos (75%) [41]. In the North Pacific region, which is geographically proximate to La Comarca Lagunera, a 74.3% prevalence was reported. In contrast, EscárcegaÁvila [47] reported a 40% prevalence in Juarez, Chihuahua, located in north-central Mexico; within the same city, a seroprevalence of 28% was documented among veterinary professionals, and a significantly higher prevalence of 51.04% was observed among canines originating from states within the northwestern region [48]. Conversely, a study conducted in La Comarca Lagunera, which involved the collection of 519 engorged adult ticks identified as *R. sanguineus*, yielded a prevalence of 10% [49].

The high prevalence in the region can be attributed to the semi-arid nature of the local climate and the high density of the tick *R. sanguineus* [3,19,25,40,50] and because, as Dantas-Torres’ [51] posits, since the vector *R. sanguineeus* exhibits a reduced cycle length under conditions of elevated humidity (35–95%) and temperature (30–35 °C), this condition is common in the study area. Another possible explanation is that the incorporation of dogs displaying clinical suspicion has been demonstrated to enhance the probability of identifying seropositive subjects, a phenomenon that is predicated within the framework of a selective screening methodology [1,52]. Furthermore, the diagnostic instrument utilised has been determined to possess elevated sensitivity and specificity, thereby ensuring the robustness of the outcomes. However, the test in question does not differentiate between active infections and those that have been previously exposed, which may result in an underestimation or overestimation of the actual values of current infection.

In order to establish strategies for the control of tick populations that encompass both canines and their environment, further research is required. To address this issue, it is possible to use chemicals through the application of various veterinary preparations, including spot-on solutions, collars impregnated with chemicals, shampoos, aerosols, baths, and powders [51]. Nevertheless, reliance on chemical compounds as the sole means of managing infestations of ticks can result in detrimental environmental consequences [53]. To optimize results, the implementation of non-chemical control methods is recommended, like the modification of the habitat of the ticks, such as the sealing of crevices and keeping grass and weeds short [51]. In addition, the biocontrol of the ticks is a viable prospect, as evidenced by recent advancements in the field, including the development of vaccines against tick-borne diseases for canine use, as highlighted by [54].

### 4.2. Clinicopathological Relationship with Suspicious Manifestations

The study revealed a significant association between seropositivity and the manifestation of clinical signs associated with ehrlichiosis, particularly haemorrhage (OR: 5.03). This finding is consistent with the pathophysiology of *Ehrlichia canis*, which affects endothelial cells and alters haemostatic homeostasis, predisposing to haemorrhagic phenomena [52,55]. The hypothesis that the presence of haemorrhage may serve as a useful clinical marker for diagnostic suspicion was statistically confirmed, thereby supporting its use as a preliminary criterion in the clinical approach, particularly in low-resource settings where adequate diagnostic tests and methods are lacking.

### 4.3. Associations with Haematological Variables (Anaemia and Thrombocytopenia)

Among the haematological variables, thrombocytopenia demonstrated the strongest association with seropositivity (OR: 43.24). This finding is consistent with the pathophysiological findings of the disease, which describe immune-mediated platelet destruction and splenic sequestration as central mechanisms in canine monocytic ehrlichiosis [30,37,39,50,52,55,56,57,58,59]. Anaemia demonstrated a significant association (OR: 4.17), indicative of a combination of persistent bleeding, chronic inflammation, and potential myelosuppression. These findings serve to reinforce the continued importance of basic haematology as a complementary tool for syndromic diagnosis [42,60].

Logistic regression analysis demonstrated that the probability of obtaining a positive diagnosis increased significantly as the absolute platelet count decreased, thereby confirming the usefulness of platelet count as a possible predictive variable. However, it is imperative to acknowledge the potential challenges in interpreting platelet counts in the context of *Ehrlichia canis* infection. Specifically, the likelihood of obtaining a positive result for *Ehrlichia canis* with a normal platelet count, or vice versa, or a negative result for *Ehrlichia canis* with a low platelet count cannot be underestimated [61,62,63].

### 4.4. Geographical Distribution (Rural vs. Urban)

Seroprevalence exhibited a marked difference according to geographical location, being significantly higher in rural areas compared to urban areas. This discrepancy is indicative of environmental conditions that are more conducive to vector proliferation in rural areas, in addition to laxer dog tenure practices and reduced access to veterinary services [2,3,41,64,65,66]. The municipalities of San Pedro and Matamoros exhibited the highest number of positive cases, a phenomenon that is likely attributable to a combination of ecological and social factors [4,25,50]. These regions could be designated as priority endemic zones for the implementation of prevention, surveillance, and community education campaigns. On the other hand, the higher prevalence of *Ehrlichia canis* in rural areas compared to urban areas could be explained by the fact that it is more common for dogs in rural areas to live outside homes, which increases exposure to vectors [67]. This finding serves to reinforce the necessity of continuous epidemiological monitoring [49,50], particularly in regions where there is a low rate of dog ownership and where socio-environmental conditions are conducive to the proliferation of this disease [1].

### 4.5. Public Health Implications and Potential Zoonoses

Although *Ehrlichia canis* is not generally regarded as a direct zoonotic agent, the presence of infected ticks poses an indirect risk to human health, given their role as vectors of zoonotic agents, such as *E. chaffeensis* or *Rickettsia* spp. [68,69].

### 4.6. Limitations and Recommendations

A key limitation of this study is the inclusion of only dogs that were clinically suspected, which restricts the generalisability of the results to the broader canine population. Furthermore, the serological laboratory test applied does not allow for differentiation between recent, chronic, or previous infections. Subsequent studies should consider extending the coverage to asymptomatic dogs in order to estimate population prevalence. In addition, it will be essential to integrate serological diagnosis with molecular tests, such as PCR. In the domain of public policy, the implementation of educational programmes at the community level is recommended, with a view to preventing vector-borne diseases. It is further recommended that public policies for dog population control and the implementation of permanent deworming programmes be promoted. In addition, the incorporation of canine ehrlichiosis as a local public health surveillance indicator is proposed.

## 5. Conclusions

A significant prevalence of *Ehrlichia canis* (63.9%) has been observed in canine specimens exhibiting suspicious clinical signs in the Laguna Region of Coahuila, suggesting active circulation of the pathogen in the area.

The disease under investigation demonstrated a robust correlation with clinical haematological variables, particularly thrombocytopenia, anaemia, and haemorrhagic manifestations. This finding serves to further substantiate its clinical profile as a valuable instrument for diagnostic suspicion in primary care settings.

The study’s findings revealed substantial disparities between rural and urban regions, underscoring the influence of social and environmental factors on the distribution of risk.

It is important to acknowledge the dual nature of canine ehrlichiosis as a veterinary health concern and as a manifestation of systemic inequities that impinge upon public health, animal welfare, and the social fabric.

The implementation of intersectoral control and prevention strategies is imperative, with a focus on epidemiological monitoring, community education, systematic deworming, and integrated management of the canine population, particularly in rural and marginalized areas.

The supplementary data could be find in File S1 Hoja 1-Erliquiosis.

## Figures and Tables

**Table 1:** Descriptive statistics for non-dichotomous social risk and public health variables relating to the owners of the dogs included in the study.

Variables Analyzed	Number of Dogs			Multivariate Analysis		
	*n*	(+)	Prevalence (%)	CI (95%)	*χ* ^2^	*p*-Value (<0.05)
Sample origin						
Francisco I. Madero	53	44	83.02	2.39–10.01	14.27	**
Viesca	10	6	60.00			
Matamoros	93	58	62.37			
San Pedro	93	48	51.61			
Highest level of schooling						
No education	6	4	66.67	0.37–10.92	3.89	NS
Elementary	6	6	100.00			
Middle school	24	14	58.33			
High school	86	54	62.79			
Higher education	127	78	61.42			

CI (95%): confidence interval (95%); *p*-value: probability; *n*: sample number; (+): positive patients; χ^2^: chi-square value; ** *p* < 0.01; NS = no significance.

**Table 2:** Descriptive statistics of the variables included in the study.

Variables Analysed	Number of Dogs			Multivariate Analysis			
	*n*	(+)	Prevalence	*OR*	CI (95%)	*χ* ^2^	*p*-Value (<0.05)
Age of pet							
Puppy (<1 year) ª	47	32	68.09	1.34	0.68–2.64	0.73	NS
Adult (≥1 year)	202	124	61.39				
Presence of ticks							
Yes ª	126	84	66.67	1.42	0.85–2.37	1.76	NS
No	123	72	58.54				
Specific signs (haemorrhagic)							
Yes ª	150	116	77.33	5.03	2.89–8.76	34.76	***
No	99	40	40.4				
Anaemia (*HCT* < 32.5%)							
Yes ª	96	78	81.25	4.17	2.28–7.61	23.10	***
No	153	78	50.98				
Thrombocytopenia (*PLT*# <117 × 10^9^/L)							
Yes ª	107	103	96.26	43.24	15.05–124.20	90.58	***
No	142	53	37.32				
Leukopenia (*WBC*# < 6 × 10^9^/L)							
Yes ª	48	30	62.5	0.99	0.52–1.90	0.00	NS
No	201	126	62.69				
Lymphopenia (*LYM*# < 0.8 × 10^9^/L)							
Yes ª	31	15	48.39	0.51	0.24–1.09	3.08	NS
No	218	141	64.68				
Owner’s residence							
Rural ª	129	91	70.54	2.03	1.20–3.41	7.12	**
Urban	120	65	54.17				

CI (95%): confidence interval (95%); OR: odds ratio (OR); *p*-value: probability; *n*: sample number; (+): positive patients; χ^2^: chi-square value; HCT: hematocrit; PLT: platelet; WBC: white blood cell count; LYM: lymphocytes; 10^9^/L: micrograms per liter; ª: dichotomous dominant variable; NS = no significance; ** *p* < 0.01; *** *p* < 0.001.

## Data Availability

The datasets used along with this research could be available from the corresponding author on reasonable request.

## Supplementary Materials

The following supporting information can be downloaded at: https://www.mdpi.com/article/10.3390/vetsci12080771/s1, File S1 Hoja 1-Erliquiosis.
